# Diet–microbiome–disease: Investigating diet’s influence on infectious disease resistance through alteration of the gut microbiome

**DOI:** 10.1371/journal.ppat.1007891

**Published:** 2019-10-31

**Authors:** Erica V. Harris, Jacobus C. de Roode, Nicole M. Gerardo

**Affiliations:** Department of Biology, O. Wayne Rollins Research Center, Emory University, Atlanta, Georgia, United States of America; Institut Pasteur, FRANCE

## Abstract

Abiotic and biotic factors can affect host resistance to parasites. Host diet and host gut microbiomes are two increasingly recognized factors influencing disease resistance. In particular, recent studies demonstrate that (1) particular diets can reduce parasitism; (2) diets can alter the gut microbiome; and (3) the gut microbiome can decrease parasitism. These three separate relationships suggest the existence of indirect links through which diets reduce parasitism through an alteration of the gut microbiome. However, such links are rarely considered and even more rarely experimentally validated. This is surprising because there is increasing discussion of the therapeutic potential of diets and gut microbiomes to control infectious disease. To elucidate these potential indirect links, we review and examine studies on a wide range of animal systems commonly used in diet, microbiome, and disease research. We also examine the relative benefits and disadvantages of particular systems for the study of these indirect links and conclude that mice and insects are currently the best animal systems to test for the effect of diet-altered protective gut microbiomes on infectious disease. Focusing on these systems, we provide experimental guidelines and highlight challenges that must be overcome. Although previous studies have recommended these systems for microbiome research, here we specifically recommend these systems because of their proven relationships between diet and parasitism, between diet and the microbiome, and between the microbiome and parasite resistance. Thus, they provide a sound foundation to explore the three-way interaction between diet, the microbiome, and infectious disease.

## Introduction

**Fig 1:**
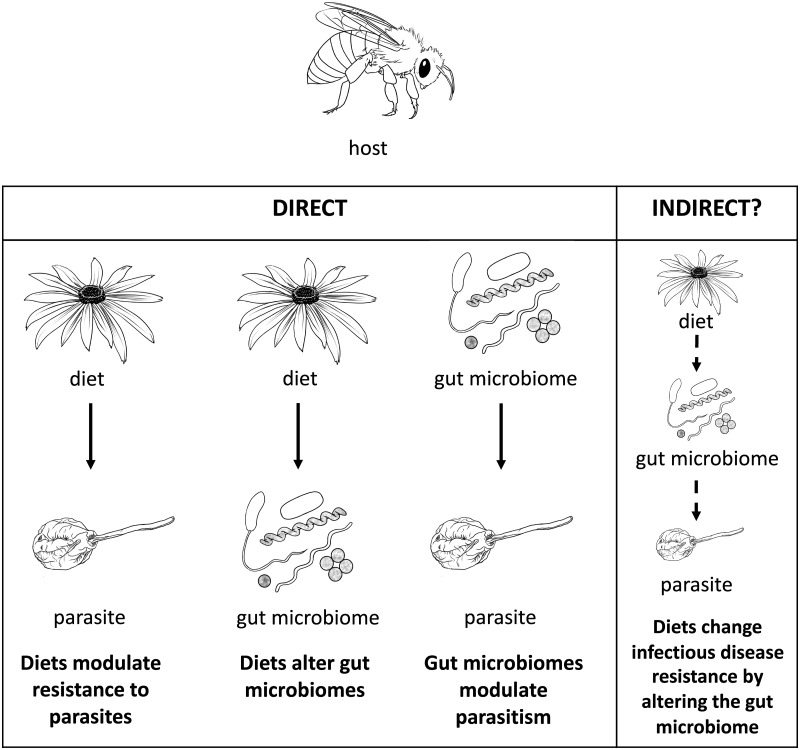
Direct and indirect relationships between host diet, host gut microbiome, and parasites. In bees, studies have independently shown that diets modulate resistance to parasites [9,21], diets alter gut microbiomes [75], and gut microbiomes modulate parasitism [90,107]. However, it is not known whether there is an indirect link between the three based on these direct relationships. Alternatively, the host immune system can indirectly alter this potential three-way interaction by modulating antimicrobial peptides or pattern recognition receptors via diet or the gut microbiome to fight parasites [11,110].

**Fig 2:**
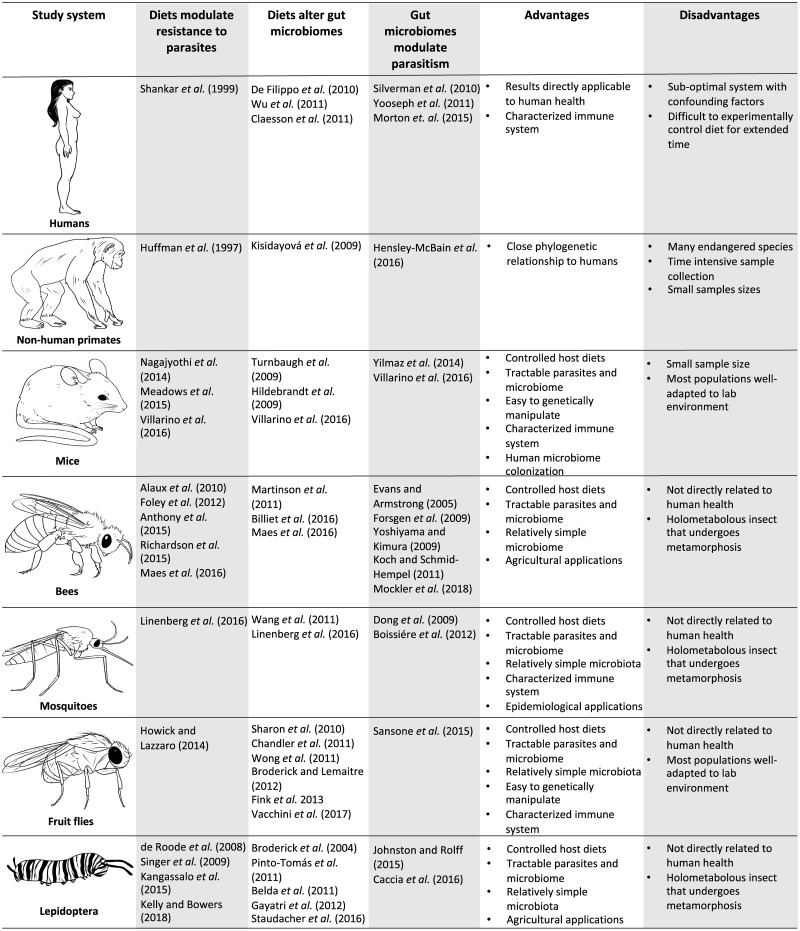
Animal systems showing three separate, direct relationships between diet, parasites, and the gut microbiome. Mice and insects are ideal systems to study the potential indirect, three-way link due to the systems’ controlled host diets, tractable and relatively simple microbiota, and tractability of parasites.

Parasites can severely reduce host fitness, and host defenses against parasites are under strong selection. Hosts and parasites are often studied as pair-wise interactions [1] without considering the environment in which they interact [2]. This is problematic because biotic and abiotic factors can have strong effects on host resistance to parasitic infection [3,4]. One increasingly recognized environmental factor that influences disease is host diet (Fig 1). Host diet also importantly shapes the gut microbiome in a wide range of hosts (Fig 2).

The gut microbiome, in turn, can be a crucial driver of infectious disease. The complex community of microorganisms inhabiting an animal’s digestive tract constitutes the gut microbiota, and their collective genetic content constitutes the gut microbiome. Changes in gut-associated microbial community composition and diversity have been associated with *Clostridium difficile* infection in humans [5] and malaria infection in mosquitoes [6].

Current understanding thus shows three important relationships: (1) diet can alter disease resistance; (2) diet can affect the gut microbiome; and (3) the gut microbiome can reduce or increase disease resistance. The potential link between these relationships remains understudied and poorly understood. Specifically, although these relationships suggest that diets could increase or reduce disease resistance by altering the host gut microbiome, there are no existing studies to support this. Instead, most studies have independently investigated the relationships between diet and disease resistance, diet and the gut microbiome, and the gut microbiome and disease resistance (Fig 1). For example, studies have shown separately that diet affects the gut microbiome and that the gut microbiome affects parasitic resistance in both mice and mosquitoes infected with *Plasmodium* spp. [7,8]. Whether this increased resistance is a result of the diet-altered microbiome is unknown. Similarly, honeybees fed aged mixed-pollen diets have an increased relative abundance of *Frischella perrara*, and these diets also increase resistance to bacterial and microsporidian parasites; whether this increased resistance is the result of a diet-altered microbiome is also unknown [9]. It is also important to note that host immunity could play a key role in directly or indirectly modulating diet–microbiome–disease interactions [10]. For example, *F*. *perrara*, the same gut microbe that is correlated with aged mixed-pollen diets, also activates the honeybee immune system [11], making it difficult to determine the sequence of events between host diet metabolism, host immunity activation, and parasitic infection inhibition. In this review, we focus on the interaction between host diet, the gut microbiome, and parasites without specific consideration of the role of host immunity in most cases. Ultimately, it would be of interest to investigate the effects of diet and the microbiome on immunity where feasible.

The potential for diet to alter infectious disease resistance by altering the gut microbiome is relevant to a wide variety of animal systems, including humans. In particular, given increasing calls to create personalized diets to augment human gut microbiomes [12], it is crucial to determine how such changes in diet will make hosts more or less susceptible to infectious disease agents. Because our focus is on infectious diseases, we define parasites as microorganisms that can cause infectious disease (bacteria, fungi, protozoa, and viruses). The goal of this review is to provide guidelines to study how diets indirectly change infectious disease resistance by altering the gut microbiome and to suggest suitable model systems to address this question. Using key references that have relevance across taxa, we begin by reviewing the aforementioned two-way relationships. We then discuss the challenges that need to be overcome to specifically integrate these separate relationships into a cohesive framework. Finally, we synthesize methods by which we can empirically test this potential three-way interaction. Our review and recommendations are not meant to be exhaustive but rather to provide a step towards advancing our understanding of how a host’s diet and gut microbiome interact to drive infectious disease resistance.

## Diets modulate resistance to parasites

Certain diets have been shown to confer protection against infectious diseases in multiple animal systems. Specifically, many animals can obtain antiparasitic diets by eating plants with toxic defensive chemicals. Nematode-infected chimpanzees, for example, eat bitter plants with nematocidal compounds [13,14], and woolly bear caterpillars infected with parasitoid flies increase their consumption of diet alkaloids, reducing infection [15]. Similarly, monarch butterfly larvae suffer less protozoan infection when feeding on milkweed plants with high concentrations of cardiac glycosides [16–19], anicia checkerspot butterflies are more immunocompetent when fed plants with higher concentrations of iridoid glycosides [20], and bumblebees that consume alkaloid-rich nectar experience reduced infection with trypanosome gut parasites [21,22]. Thus, many herbivores exploit plant defensive chemistry to reduce parasite infection and growth.

Animals can also increase parasitic resistance by increasing the quality and types of foods that they eat. For example, honeybees with a diverse pollen diet are more immunocompetent than individuals fed on a monofloral diet [23]. Similarly, lab-reared honeybee larvae gain resistance to fungal pathogens when nutrient-poor diets are supplemented with polyfloral pollens [24]. Fruit flies fed low-sugar diets have lower bacterial pathogen load and reduced mortality than when fed on high-sugar diets [25]. Mice infected with protozoan parasites that cause Chagas disease have reduced parasitemia when fed high-fat diets [26]. As with other animals, the diet of humans is a strong driver of parasite infection. Human malnutrition is a global concern that is associated with micronutrient deficiencies and is linked to immunodeficiency. For example, malnourished children in Papua New Guinea are at higher risk of malaria infection. Supplementing their diets with vitamin A reduces both *Plasmodium falciparum* density and disease symptoms, including fever [27].

Diets can have a complex effect on a host’s ability to fight infection. The addition of a dietary component may not always positively correlate with parasitic resistance; the effect of diet on parasites can be negatively correlated, with an increase in dietary components being correlated with a decrease in parasitic resistance. For example, mice infected with protozoan parasites that cause murine malaria and fed folate-supplemented diets have decreased survival and decreased resistance compared with mice fed the standard dose of recommended folate [28]. Similarly, greater wax moths infected with a fungal parasite and fed high-nutrition diets were more susceptible and experienced a higher mortality rate than infected individuals raised on low-nutrition diet [29].

Thus, diets can confer protection against infectious diseases by direct interference through chemical inhibition of parasites or modulation of available resources to fight pathogens. Alternatively, diets may confer protection through alteration of microbial competition, which until recently has been largely overlooked and which we will address next.

## Diets alter gut microbiomes

As with other ecological communities, gut microbial communities are groups of interacting species that occur together at the same time in a defined place. Recent technological advances have increased the feasibility of studying gut community composition and function [30,31]. Gut microbial communities have a structure that is characterized by species richness (the number of species), species evenness (the relative abundance of each species), and species diversity (a metric accounting for both species richness and evenness). Because different microbial species can have diverse roles, the overall function of these communities is typically characterized by assaying total genetic content (metagenomics) and gene expression (transcriptomics).

Different host species have different microbiomes driven by host genetics, evolutionary history, and evolved dietary specialization [32–34]. Termites, for example, are consumers of cellulose-based plant materials but cannot directly break down cellulose; instead, they harbor vertically transmitted microbial gut symbionts—bacteria, protists, and archaea—that contain cellulose-digesting genes [35]. Termites that specialize in different feeding groups (e.g., wood, grass, humus, soil, and fungus) harbor significantly different assemblages of gut microbes [36], a signature of evolved microbiome specialization.

The microbiome, however, is also plastic, and changes in diet can alter gut microbial community composition [37,38] and thus have the potential to importantly shape community function. For example, in wood-feeding termites, changes in diet are accompanied by shifts in the dominance of protist species [39]. In humans, major shifts in diet (i.e., shift from high-fat/low-fiber to low-fat/high-fiber diet) also significantly influence gut community composition over short time periods [38,40]. However, the human gut microbiome is relatively stable over time [40,41], with long-term diet strongly correlating with bacterial enterotype, the classification of microbiome samples based on clustering in ordination analyses [37,42,43]. After a dietary perturbation, communities tend to shift back towards their original community composition and stabilize. Although such plastic changes of the gut microbiome in response to dietary shifts have been observed across the animal kingdom [44–49], it is not clear whether diets change the microbiome through similar mechanisms across systems and whether these changes are generally stable or transient.

Supplements added to the diet can also modulate the gut microbiota. Prebiotics are dietary supplements that, once consumed by the host, act as food or substrates for the host microbiota. More specifically, the “prebiotic effect” is the selective stimulation of growth and metabolic activity of a single or limited number of taxa in the gut microbiome that confers health benefits to the host [50,51]. A common prebiotic for humans is inulin and its chemical derivatives [52]. Inulin is a soluble fiber found in many plants naturally occurring in foods, such as chickory root, garlic, and onions [53], and is also commercially produced. In clinical studies, healthy humans administered inulin-containing foods over the span of weeks show a change in microbial community composition, with significant increases in *Bifidobacteria* [54,55]. In turn, *Bifidobacteria* and *Lactobacilli* are common genera used as probiotics in several hosts [7,56]. Probiotics are non-native live microorganisms orally consumed by hosts and beneficial to host health. Probiotics naturally occur in fermented foods such as yogurt. The combined synergistic effect of prebiotics and probiotics is synbiotics [57]. *Bifidobacteria* and *Lactobacilli* bacteria may play a role in the treatment or prevention of several human infections, including the infection of the human digestive tract caused by *C*. *difficile* and human vaginal bacterial infections [58,59]. However, it can be difficult to elucidate the efficacy and mechanisms of prebiotics and probiotics in humans. Interestingly, use of prebiotic and probiotic supplements in more tractable model systems, such as bees, shrimp, and fish, suggests that such supplements can confer antimicrobial activity, increase immune gene expression, and decrease the load of bacterial pathogens and intestinal parasites in these systems [60–62].

A major issue with elucidating the effects of diet on the human gut microbiome is the occurrence of confounding factors. For example, human children from rural Africa and modern Western Europe fed on plant- and animal-based diets, respectively, exhibit significant differences in bacterial communities: *Prevotella*, *Xylanibacter*, and *Treponema* genera are abundant in rural Africans but absent in Western Europeans [45]. The bacteria in these genera contain genes involved in cellulose hydrolysis and are associated with the capacity to metabolize indigestible polysaccharides commonly found in plants. Despite the apparent link between diet and microbiota composition, factors other than diet, such as host genetics, race, ethnicity, variation in antibiotic use, and geographically varying environmental factors, could also play a role. Human microbiome research is also hampered by logistical constraints, such as inconsistent self-assessments on dietary questionnaires and budget limitations that prevent supplying large cohorts with controlled diets for an extended period of time [44,63]. Ironically, what this means is that, despite the fact that human health is the primary focus of diet–gut microbiome research, humans are a suboptimal system to understand how diet shapes microbial community dynamics. Therefore, to better understand the mechanistic links between diet and the gut microbiome, it is beneficial to study systems in which confounding factors can be more easily controlled [64–67].

Mice are the most common animal model used to translate gut microbiome research to human health, in part because human fecal microbial communities can successfully colonize germ-free, inbred mouse strains [44]. Major dietary shifts from low-fat/high-fiber to high-fat/high-sugar diets in such mice cause rapid changes in microbial community structure and function [44,68]. Thus, as with humans, diet is a major driver of microbiome composition in mice.

Insects also provide excellent systems to study the effects of diet on the gut microbiome [64]. Similar to termites, mentioned previously, microbial communities of fruit fly species vary with the different fruits and flowers on which these species are specialized to feed. Fly microbial communities are also plastic, changing with dietary shifts [69]. For example, within a single population of the fly *Drosophila elegans*, feeding on two different flowering plant genera results in different abundances of the dominant bacterial families. Similarly, feeding *Drosophila suzukii* fruit-based natural and nonfruit artificial diets results in altered communities [70]. Diet also influences *Drosophila melanogaster* gut microbial community composition [71–73]. For example, altering fat content, particularly from high fat to no fat (i.e., starvation), of *D*. *melanogaster* diet results in changes in the abundance of some bacteria as well as changes in the overall number of microbes in the community [74].

Diet also strongly influences microbial communities of bees, butterflies, and moths. Bee gut microbial communities are dominated by eight dominant bacterial phylotypes (bacterial clusters based on sequence similarity) that can be modified with alternative syrup and pollen diets [33,75]. Similarly, the dependence of gut microbial community composition on alternative larval host plants is widespread in lepidopteran species [76–79]. For example, tobacco budworm larvae fed three alternative host plants have significantly different bacterial families [76], and there is variation in bacterial phylotypes in the gypsy moth microbiome based on alternative plant diets [80]. Although these examples demonstrate that diet affects the gut microbiome in many animal systems, the mechanisms by which this occurs are largely unknown (Box 1).

**Box 1. Crucial considerations in the study of diet–microbiome–disease interactions**

1. **Comparing microbial communities**. A major challenge plaguing the field of microbiome research is defining what variation to quantify and what variation matters [30,111,112]. Although it is becoming relatively simple to characterize a gut microbial community, it is more difficult to conclude what variation between experimental groups is biologically significant. Differences that may impact host phenotypes may lie in the presence and diversity of the microbial community, the presence of particular taxa, the abundance of particular taxa, or microbial gene expression, regardless of the genome of origin. Technological approaches vary in the degree to which they can characterize these differences. Furthermore, in the case of differences at the taxonomic level, studies define community composition differently at the phylum (Wu and colleagues, 2011), genus [45], species [113], and strain [114,115] levels. This inconsistency demonstrates that there is no workbook for which of these to quantify, requiring a thorough investigation of each system studied.
2. **Accounting for individual microbiome variation**. There is substantial individual variation in gut microbiome composition, which may be due to genetics, abiotic or biotic factors, or stochasticity. Furthermore, gut microbial communities change over development, with sometimes high species turnover, adding more variation to an animal system [116,117]. Because of the many sources of microbiome variation, studying the link between diet, the microbiome, and disease can be difficult, as the microbiome may vary for reasons other than diet. Thus, the key is to determine the relevant variation due to changes in diet and to determine how those particular changes correlate with disease resistance.
3. **Defining which dietary components influence the microbiome and disease susceptibility**. Diets have many components. Therefore, it is imperative that studies first clearly define which dietary component(s) or dietary supplement(s) are considered when assessing the influence of diet on disease resistance or on the microbiome. To date, several different dietary components have been implicated in influencing the gut microbiome in animals, including fiber, protein, plant secondary metabolites, types of fat, foodborne bacteria, and prebiotics [12,45,118]. The dietary component(s) of interest may be nutritious or toxic, depending on the system [119]. If a dietary shift is observed to modulate the gut microbiome or disease resistance, then the exact nature of what components of that diet are shifting should be characterized. Systems in which diet can be experimentally manipulated are ideal, as controlled diets eliminate confounding dietary variables, making it possible to observe the direct effect of a single dietary component on gut microbiota composition and on disease susceptibility. Furthermore, such diets can be standardized, providing the opportunity for comparisons across studies. However, one drawback of such controlled diets is that they are not generalizable to natural diets [111]. Coupling a chemically well-defined diet with a natural diet in animal systems should provide novel insights as to diet’s role in altering the microbiome and disease [47].

## Gut microbiomes modulate parasitism

Microbial symbionts, microbes that form a long-term association with hosts, can play important roles in animal health, particularly in mitigating infectious diseases. For example, aphids harbor non-gut-associated bacterial symbionts that protect them against fungal pathogens and parasitoid wasps [81,82]. Similarly, beewolf wasps incorporate symbiotic bacteria into their larval cocoons for protection against pathogenic fungi [83,84], and salamanders have skin bacterial symbionts that produce antifungal metabolites against chytrid fungus [85]. It is now clear that gut-associated microbial symbionts can also play major roles in infectious disease dynamics, with changes in microbial community structure and function being correlated with parasite infection in several systems. These community structural changes can be caused by dysbiosis (or disruption of the “healthy” microbiome) or parasite infection. Although both states have the potential to shift parasite resistance, their mechanisms can be different. In the case of dysbiosis, gut pathogens may exploit an empty niche or host physiological stress to successfully colonize the gut. Systemic parasites may exploit organism stress to disseminate and replicate throughout the body. A well-known example is microbial-conferred protection against the bacterium *C*. *difficile*, which is a leading cause of chronic diarrhea following the long-term use of antibiotics in humans. Antibiotic-induced disturbance of the gut microbial community favors the increased growth of *C*. *difficile* and recurrent infection. Clinical microbiome transplants via feces (i.e., fecal transplants) from healthy donors can be used to treat the disease in infected recipients by restoring the gut community [86–89]. Hence, *C*. *difficile* infection exploits dysbiosis by proliferating in the gut bacterial community and shows that community composition and potentially the number of bacteria are crucially important in affecting parasite invasion success. Similarly, sterile sugar–fed and antibiotic-treated bees suffer increased trypanosome infection relative to bees with a complete gut microbiome, and fecal transplants restore the bees’ gut microbiota and increase resistance [90]. Although the protective effect of the gut microbiome against parasites is evident in these and other systems, the properties of the microbiome that reduce parasitism are rarely known.

**Fig 3:**
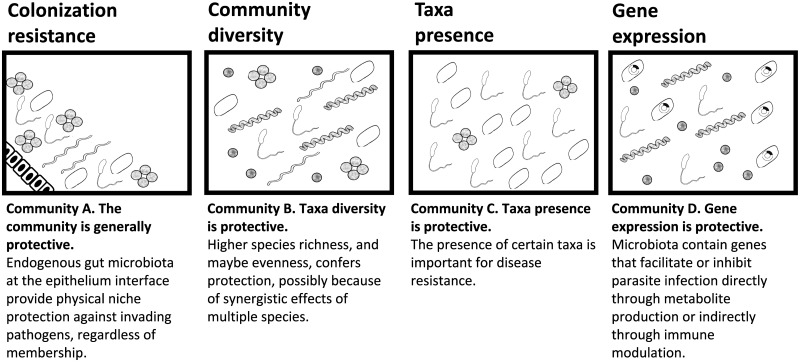
Properties of the gut microbiome that could reduce parasitism. The protective effects of the gut microbiome may derive from colonization resistance, the abundance and evenness of one or more species at various taxonomic levels, the presence or absence of particular species, or the presence or abundance of certain genes. These scenarios are not exhaustive nor mutually exclusive [107].

The protective effects of the gut microbiome may result from the presence and diversity of the microbial community, the presence of particular taxa, or the presence of particular genes within the microbial community (Fig 3). Several examples illustrate the importance of the community. As mentioned previously, the gut microbiome of bees provides protection against trypanosome infection [90,91]; however, consumption of a single bacterial class does not reduce trypanosome burdens. Similarly, a diverse bee gut community is also protective against the bacterial pathogen *Paenibacillius larvae*, the causative agent of American foulbrood [92–94]; although 11 isolated, cultured bacterial phylotypes differentially inhibit the growth of parasite strains in vitro, only the microbial cocktail of all 11 bacterial phylotypes completely inhibits the growth of *P*. *larvae* in vitro and in vivo. Desert locusts also have decreased pathogen colonization with increased numbers of gut bacterial species [95]: specifically, the presence of 2 and 3 bacterial species provides more protection against *Serratia marcescens* than the presence of only 1 species. The importance of the microbial community may result from the complementary and synergistic antiparasitic effects of different microbes. Although the benefits of a diverse microbial community are widely accepted, the mechanisms of protection are poorly understood in animal models [96,97]. Potential mechanisms include high functional diversity [98], increased functional redundancies [99], and metabolic cross-feeding [96,100].

The presence of particular taxa can also be a protective property of the gut microbiome. Several malaria-associated studies across animal systems find a correlation between particular bacterial taxa and *Plasmodium* infection. Malian children with a lower risk of infection by the malaria parasite *P*. *falciparum* have a higher proportion of *Bifidobacterium* and *Streptococcus* genera compared with higher-risk individuals [101]. This example, like others, merely presents a correlation between the presence and absence of particular gut bacteria taxa and parasites [102,103]. Demonstrating a causative link, in *Anopheles* mosquitoes, the ingestion and colonization of *Chromobacterium* results in induction of immune genes and decreased susceptibility to *P*. *falciparum* infection and dengue virus [104]. Similarly, antibiotic-treated mice inoculated with a cultured microbial cocktail containing *Bifidobacterium* and *Lactobacillus* display a decreased malaria parasite burden compared with control mice, suggesting that these taxa have a modulatory effect on parasitism [7].

Sometimes it is not the presence of the gut microbial community or presence of particular taxa in that community but rather the expressed genes of the community that have a modulatory effect on protection. For example, laboratory mice can harbor gut bacteria that express glycan surface proteins. These glycan surface proteins elicit glycan-specific antibodies that attack *Plasmodium* spp. during transmission from *Anopheles* mosquitoes to mice lacking the glycan surface protein gene [105]. Similarly, mice colonized with *Bifidobacterium breve* bacteria expressing exopolysaccharides have significantly less colonization and persistence of a murine bacterial pathogen compared with mice without bacteria expressing an expolysaccharide gene [106]. The protective effect of *B*. *breve* is linked to a gene cluster responsible for the expression of exopolysaccharides. These two studies demonstrate that protection can be induced, or pathogenesis inhibited, by manipulating the gene expression of gut microbes. Importantly, given that bacteria can horizontally transfer genes, protection against parasitic infection conferred by expressed genes has the potential to persist in a microbial community independent of the presence of particular taxa. However, these scenarios are not exhaustive nor mutually exclusive. For example, in bees, high community diversity, high bacterial abundance, and taxa presence all contribute to protection against a trypanosome parasite [107].

## Experimental approaches to study diet–microbiome–disease interactions

As is clear from the previous examples, diets alter both parasite resistance and gut microbiomes in a range of animals. Because the microbiome is an important driver of parasite resistance, these relationships suggest that diets may change parasite resistance through their effects on the gut microbiome. However, to our knowledge, the effect of the diet on infectious disease susceptibility through their impact on the microbiome has not been unequivocally demonstrated in any system. Nonhuman animal systems that have separately demonstrated that diet alters resistance to parasites, diet alters the gut microbiome, and the gut microbiome alters parasitism are ideal systems to empirically test for the potential of diet altering disease resistance by modulating the gut microbiome. To fully explore this link, researchers must study diet, the microbiome, and disease in tandem in a controlled, experimental setting. The best case studies, based on current literature, appear to be experimentally tractable insect and mouse systems (Fig 2, Box 2).

**Box 2. Case study: Composition of gut microbial community modulates severity of malaria in mice**

One study on mouse malaria investigated the three relationships that are the focus of this review: (1) diet alters disease resistance; (2) diets alter gut microbiomes; and (3) gut microbiomes modulate disease resistance [7]. This study first found that genetically inbred mice (C57BL/6) infected with *Plasmodium* significantly differed in parasite burden based on mouse vendor source. Mice from Jackson Laboratory (Jax) and Taconic (Tac) had a significantly lower number of parasites and no mortality compared with National Cancer Institute/Charles River (NCI) and Harlan (Har) mice. To test whether diet increases resistance to malarial infection, Jax (resistant) and NCI (susceptible) mice were fed two commercial chow diets: NIH-31 and Teklad 22/5. Although parasitemia was not affected in susceptible NCI mice fed these diets, the Teklad 22/5 diet significantly increased parasitemia and mortality in resistant Jax mice compared with the NIH-31 diet. This study also demonstrated that the alternative diets affect the gut microbial community composition: Jax mice fed the Teklad 22/5 diet had lower relative abundance of the bacterial family Peptostreptococcaceae compared with Jax mice fed the NIH-31 diet. The researchers then used fecal transplants, microbial supplementation, and immune assays to demonstrate that the gut microbiome reduces parasitism. However, instead of carrying out this study with mice of similar origin fed on alternative diets, the researchers used mice that varied in resistance due to different vendor origin (Jax and Tac versus NCI and Har). Thus, while suggestive of an indirect link, this study did not yet unequivocally demonstrate that diets altered disease resistance by modulating the gut microbiome.

The need to study diet, the microbiome, and disease together is clear when one tries to connect the three across separate studies. For example, bee studies have shown that diets rich in alkaloids increase resistance to a variety of parasites, including trypanosomes, fungi, and microsporidia [9,21,24]. Separate studies have shown that pollen supplemented with nectar diet alters the gut microbial community composition of bee larvae [75], and, as highlighted previously, other studies have shown that bee gut microbes can increase resistance to pathogens and parasites [90,92]. Complications arise when trying to link these studies. First, the dietary components considered were different across studies (Box 1). Second, the two-way relationships were studied in different life stages: while the effect of alkaloid diets on the trypanosome *Crithidia bombi* was investigated in bee adults [21], and the effect of gut microbes on *C*. *bombi* was also studied in adults [90], the effect of protein and sugar-rich diets on bee microbial communities was investigated in bee larvae [75]. Similarly, in mosquitoes, a particular larval aquatic diet increases resistance to *Plasmodium* spp. and also increases the relative abundance of two bacterial families [8]. Separate studies have demonstrated that mosquito gut microbes reduce parasitism with *Plasmodium* [6,104]. Ideally, researchers would study all three interactions across life stages, because diet differences across immature stages could have lifelong effects on adult individuals. Similar to bees, the dietary components and life stages between these mosquito studies were different—fish flakes for aquatic larvae versus sugars and blood in adults. We are aware of only a single study using mice in which all three separate components were considered [7]. However, even within this study, it is not clear that diet mediated its antiparasitic effects by modulating the gut microbiome (Box 2).

In order to study the potential effect of diet-altered protective gut microbiomes on infectious disease, we propose several recommendations. First, studies should only use animal systems in which host diet increases resistance to tractable parasites. Second, host genetics should be carefully controlled. Ideally, host genetics can be controlled by testing individuals with identical or similar genetic backgrounds, such as monozygotic twins or full siblings. Alternatively, unrelated individuals can be partitioned across treatments to reduce confounding factors. Third, it is important to determine whether alternative diets are associated with alterations of the microbial community and if so, to try and elucidate whether the same dietary components are responsible for altering parasite resistance. Fourth, it should be demonstrated directly, through a unified study, that gut microbial community variation caused by alternative diets correlates with disease susceptibility. A particularly powerful approach for this test is to use fecal transplants [7,44,90]. Beyond demonstrating that the actual altered microbiome provides resistance to parasites, carefully manipulated fecal transplants can also be used to elucidate whether the presence of the entire gut community is needed for protection or whether the presence and abundance of particular taxa are more important. To tease apart the protective mechanism of species presence and interactions, cultivated microbial transplants of specific community members have been effective in bees, mosquitoes, and mice [7,92,104]. Silencing microbial community members’ genes is also an effective way to resolve whether the presence and expression of certain genes are responsible for the protective mechanism of the gut microbiome, as shown in mice [105,106]. Finally, for animal systems with robust genetic tools, including mosquitoes and moths, both host immune genes and microbiome toxin genes can be silenced to determine their interplay [6,108,109].

## Conclusion

Existing studies suggest that diets can alter host resistance to parasites by modulating the gut microbiome, but conclusive studies remain lacking. Although an understanding of diet–microbiome–disease interactions is critical for humans, we propose alternative animal model systems to test fundamental properties of this potential interaction. These animals are relevant to agriculture and epidemiology, and they allow for carefully controlled experiments with few constraints on sample size. Most importantly, they are tractable systems that have strong evidence of each separate interaction: diets modulate resistance to parasites, diets alter gut microbiomes, and gut microbiomes modulate parasitism (Fig 2). Existing experimental tools now allow researchers to build on the separate, direct relationships to determine whether there is an indirect link between host diet, host gut microbiome, and parasite infection. Elucidation of the importance and ubiquity of such a link will help us better understand the therapeutic potential of diets and gut microbiomes to control infectious disease.
